# Machine learning in neuroimaging for predicting H3K27M mutations in diffuse midline gliomas: a systematic review and meta-analysis

**DOI:** 10.3389/fmed.2026.1746840

**Published:** 2026-03-25

**Authors:** Hongfei Wang, Shiyu Chang, Weixiang Wang, Mingyu Zhang, Yunqian Li

**Affiliations:** 1 Department of Neurosurgery, The First Hospital of Jilin University, Changchun, China; 2 Department of Gastroenterology, The First Hospital of Jilin University, Changchun, China; 3 Department of Thyroid Surgery, The First Hospital of Jilin University, Changchun, China; 4 Department of Breast Surgery, The First Hospital of Jilin University, Changchun, China

**Keywords:** artificial intelligence, diffuse midline gliomas, H3K27M mutations, meta-analysis, neuroimaging

## Abstract

**Objective:**

We aimed to evaluate the diagnostic performance of neuroimaging-based machine learning (ML) models for non-invasive prediction of H3K27M mutations in diffuse midline gliomas (DMG).

**Methods:**

Following PRISMA-DTA guidelines, we searched four databases up to May 2025 to identify eligible studies evaluating neuroimaging-based ML for predicting H3K27M mutations in DMG. Study quality was assessed using PROBAST+AI and GRADE. Bivariate random-effects models were used to pool sensitivity, specificity, and area under the receiver operating characteristic curve (AUC).

**Results:**

Sixteen studies were included, comprising 2,357 patients in internal validation cohorts and 1,792 patients in external validation cohorts. MRI-based ML models showed strong diagnostic performance in internal validation, with pooled sensitivity of 0.86 (95% CI: 0.79–0.91), specificity of 0.82 (95% CI: 0.75–0.87), and AUC of 0.91 (95% CI: 0.88–0.93). For PET/CT–based ML models, pooled sensitivity was 0.58 (95% CI: 0.44–0.71) and pooled specificity was 0.65 (95% CI: 0.46–0.81), with an AUC of 0.61 (95% CI: 0.57–0.66). MRI-based ML showed significantly higher sensitivity (*Z* = 3.71; *P* < 0.01) and AUC (*Z* = 11.42; *P* < 0.01) than PET/CT-based ML. Subgroup analysis indicated that MRI-based deep learning (DL) models outperformed conventional machine learning (cML) algorithms (*P* = 0.01). Models using DNA sequencing as the reference standard showed higher specificity than those using immunohistochemistry (*P* < 0.001).

**Conclusion:**

MRI-based ML demonstrates high accuracy and generalizability for non-invasive H3K27M prediction in DMG, seemingly outperforming current PET/CT-based ML. The adoption of DL architectures and DNA sequencing as the reference standard may further improve performance, supporting the clinical utility of MRI-based ML for molecular stratification.

**Systematic review registration:**

https://www.crd.york.ac.uk/prospero/display_record.php?RecordID=1078673, identifier CRD420251078673.

## Introduction

Diffuse midline glioma (DMG) is a leading cause of pediatric central nervous system (CNS) cancer-related death. According to the 2021 WHO classification, this entity is designated as WHO grade 4 and classified molecularly as a “Diffuse midline glioma H3 K27-altered” (1). Approximately 80% of cases harbor a canonical H3.3 or H3.1 K27M mutation (2, 3). DMG primarily affects children aged 4–10 years and is associated with a dismal median overall survival of only 9–11 months, largely due to profound resistance to standard chemoradiotherapy (2, 4). However, emerging therapies offer new promise. Phase II trials have demonstrated that the H3K27M-targeted agent dordaviprone (ONC201) prolongs median overall survival to 21.7 months when administered after initial radiotherapy (5, 6). Consequently, early and non-invasive prediction of the H3K27M mutation status is crucial for guiding personalized therapy and improving patient outcomes (5, 7, 8).

Despite their integral role in clinical management, conventional diagnostic modalities for DMG—broadly categorized into invasive approaches (e.g., histopathological biopsy and lumbar puncture-based cerebrospinal fluid (CSF) analysis) and non-invasive imaging [including magnetic resonance imaging (MRI) and positron emission tomography/computed tomography (PET/CT)]—have important limitations (1, 9–12). Invasive methods carry procedural risks, with biopsy not feasible in all anatomically sensitive midline locations and CSF-based liquid biopsy demonstrating low sensitivity for H3K27M mutations at initial presentation (12, 13). Conversely, non-invasive imaging, while safer, is subject to inter- and intra-observer variability, and its diagnostic accuracy is limited by overlapping imaging features between mutant and wild-type tumors (14–18). Notably, conventional visual assessment of these images struggles to extract these quantitative radiomic features. This inability to decode imaging data represents a key limitation, as these features may harbor critical molecular signatures (16, 17, 19).

This fundamental limitation has spurred the development of neuroimaging-based machine learning (ML) models to decode these imaging signatures associated with tumor genotype. Broadly, these approaches can be conceptually divided into two main methodologies. Radiomics-based prediction typically employs conventional machine learning (cML), in which engineered quantitative features are extracted from regions of interest and used to train classifiers. In contrast, deep learning (DL)-based prediction utilizes architectures like convolutional neural networks (CNNs) to automatically learn hierarchical feature representations directly from the imaging data, potentially capturing more complex patterns. Neuroimaging-based ML approaches, encompassing both cML and DL strategies, have shown promise for non-invasive prediction of molecular subtypes in gliomas (17, 20–23). By integrating quantitative features that are not perceptible to conventional imaging (24), these models may enable early, patient-specific molecular characterization. Nonetheless, heterogeneity in imaging sequences, algorithmic choices (cML vs. DL), and external validation cohort sizes across studies contributes to variable diagnostic performance (25, 26), raising concerns about generalizability.

This systematic review and meta-analysis therefore aimed to synthesize and compare the diagnostic performance of MRI- and PET/CT-based ML models for predicting H3K27M mutation status in DMG, and to evaluate methodological factors associated with model performance and generalizability.

## Materials and methods

This meta-analysis was conducted in strict accordance with the Preferred Reporting Items for a Systematic Review and Meta-analysis of Diagnostic Test Accuracy Studies (PRISMA-DTA) guidelines (27). The full PRISMA checklist is provided in Supplementary Table 1. The protocol for this review was prospectively registered in PROSPERO (CRD420251078673).

### Search strategy

A comprehensive literature search was conducted in four major electronic databases: PubMed, Embase, Cochrane Library, and Web of Science. The search was last updated on 10 May 2025. Two independent reviewers (H.W. and S.C.) screened the titles and abstracts of all identified records, followed by full-text assessment for eligibility according to predefined criteria. The search strategy was constructed using a combination of three key thematic components: (1) ML-related terms (e.g., “artificial intelligence,” “machine learning,” “deep learning”); (2) terms specific to the molecular target (e.g., “H3K27M mutation”); and (3) disease-related terms (e.g., “diffuse midline glioma,” “DIPG”). Both free-text terms and Medical Subject Headings (MeSH) terms were used to ensure comprehensive coverage. No restrictions were applied regarding language or year of publication at the initial search stage. The complete and detailed search strategies for each database are provided in Supplementary Table 2. In addition, the reference lists of all included studies were manually searched to identify any further relevant publications.

### Inclusion and exclusion criteria

Inclusion criteria were determined according to the PITROS framework in Supplementary Table 3. Studies were excluded if they: (a) were reviews, case reports, conference abstracts, meta-analyses, or letters to the editor; (b) were non-English with no accessible English translation; (c) did not apply ML-based algorithms for neuroimaging; (d) did not report H3K27M mutation status; or (e) did not provide sufficient data on sensitivity, specificity, or AUC values. Initial screening and full-text evaluation were independently conducted by two reviewers (S.C. and W.W.), with duplicates identified using EndNote and manual verification. Discrepancies were resolved through discussion, and a third reviewer (M.Z.) was consulted in cases of unresolved disagreement.

### Quality assessment

Methodological quality and risk of bias were assessed using the PROBAST+AI tool (28), an updated version superseding PROBAST 2019. The PROBAST+AI tool evaluates studies across two stages: model development and model evaluation. Each stage encompasses seven domains addressing key methodological aspects, including participants and data sources, predictors, outcome evaluation, and analysis. Within each domain, the risk of bias is categorized as low (L), high (H), or unclear (U). This categorization is determined by responses to specific signaling questions. The complete list of signaling questions was provided in Supplementary Tables 4, 5. To maintain objectivity and analytical rigor, two reviewers (H.W. and S.C.) independently applied the PROBAST+AI tool to each eligible study. Any discrepancies in assessments were resolved through discussion and consensus-based resolution.

### Certainty of evidence

The certainty of evidence for pooled sensitivity and specificity estimates was evaluated using the Grading of Recommendations, Assessment, Development, and Evaluations (GRADE) framework (29). The GRADE approach systematically considers five key domains: risk of bias, indirectness, inconsistency, imprecision, and publication bias. Detailed procedures and domain-specific criteria are described in Supplementary Table 6.

### Data extraction

Two independent reviewers (H.W. and W.W.) extracted data from all eligible full-text articles. Discrepancies in extraction were resolved through consensus. The extracted data encompassed the following categories: (a) Study and patient characteristics: first author, year of publication, country, study design, location of DMG, reference standard used for H3K27M mutation diagnosis, analysis method, total number of patients analyzed, and number of patients with H3K27M mutation; (b) ML methodology: data source, ML approach, ML model, data splitting method, type of neuroimaging data; (c) Imaging acquisition parameters: scanner modality, magnetic field strength, specific MRI sequences utilized; (d) Image analysis details: scanner modality (system), feature extraction method and software, regions of interest, evaluation time, magnetic field strength; (e) Diagnostic performance data: number of true positives (TP), true negatives (TN), false positives (FP), and false negatives (FN)

Since most studies did not directly report full diagnostic contingency tables (TP, TN, FP, FN), we reconstructed these using two approaches: (1) When sensitivity, specificity, number of H3K27M-positive cases, and total sample size were available, standard formulas were used to derive TP, FP, FN, and TN. (2) For studies reporting only ROC curves, we extracted coordinates using GetData Graph Digitizer (version: 2.26.0.20) software and selected the point with the highest Youden Index to estimate sensitivity and specificity.

### Outcome measures

The primary outcome measures for assessing the diagnostic performance of the ML models were sensitivity, specificity, and the area under the receiver operating characteristic curve (AUC), as reported for both internal and external validation datasets. Sensitivity (true positive rate) was defined as the proportion of patients with H3K27M mutation correctly identified by the ML model: Sensitivity = TP/(TP + FN). Specificity (true negative rate) was defined as the proportion of patients without H3K27M mutation (wild-type) correctly identified by the ML model: Specificity = TN/(TN + FP). The AUC provided a summary measure of the model’s overall discriminatory ability across all classification thresholds. It represents the area under the ROC curve, which plots sensitivity (true positive rate) against 1 – specificity (false positive rate).

For studies reporting results from multiple independent datasets with non-overlapping patient populations, outcome data from each dataset were extracted and analyzed separately. To comprehensively compare the diagnostic performance across different algorithms, data from all eligible, distinct ML models within each study were extracted and included in the analysis. We acknowledge that multiple models from the same study were validated on the same patient cohort, leading to non-independence at the data level.

### Statistical analysis

A bivariate random-effects model was employed to perform meta-analyses (30). This model jointly synthesizes sensitivity and specificity while accounting for their inherent correlation and between-study heterogeneity. Summary receiver operating characteristic (SROC) curves were generated to depict the overall diagnostic accuracy, accompanied by 95% confidence regions (reflecting the precision of the pooled estimate) and 95% prediction regions (indicating the expected range of sensitivity and specificity for a future study). Differences in pooled diagnostic performance metrics between internal and external validation sets, as well as between PET/CT-based and MRI-based ML methods, were assessed using the Z-test.

Heterogeneity was assessed using the Higgins’ I^2^ statistic (31) and further explored through pre-specified subgroup analyses, meta-regression and bivariate boxplots. These analyses were conducted separately for internal and external validation sets (32). The distribution of ML algorithms across included studies was visualized via radar plots. Fagan nomograms were constructed to estimate clinical utility by illustrating post-test probabilities of H3K27M mutation for various pre-test probabilities. Publication bias was evaluated using Deeks’ funnel plot asymmetry test by regressing the log diagnostic odds ratios against effective sample sizes (33). Meta-analyses were performed using the midas and metadta packages in Stata version 18, while quality assessments and radar plots were generated using R version 4.3.1. A two-sided *P* < 0.05 was considered statistically significant for all analyses.

## Results

### Study selection

**FIGURE 1:**
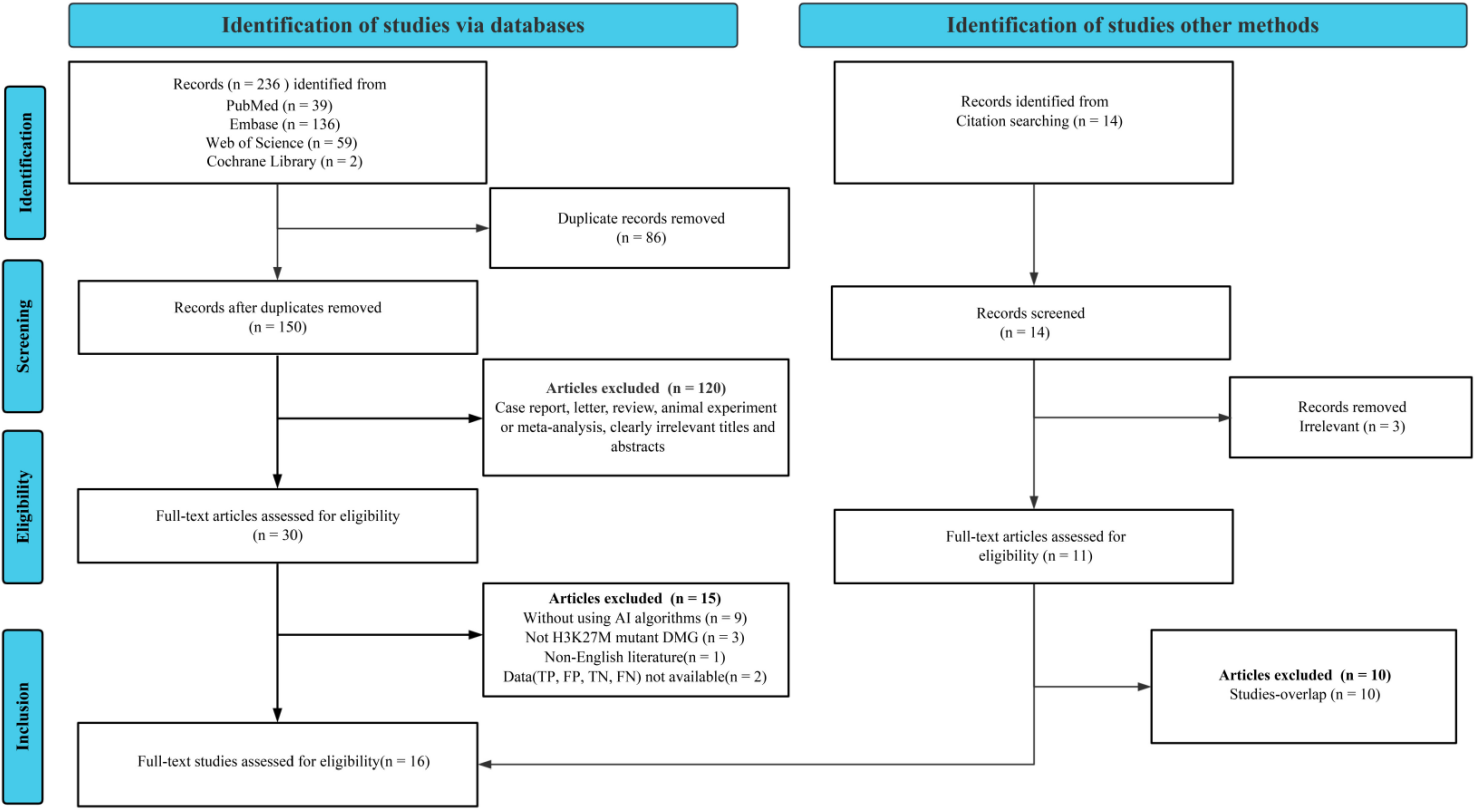
PRISMA flow diagram illustrating the study selection process.

A total of 236 potentially relevant records were identified through the initial database search. Following the removal of 86 duplicates, 150 unique records underwent title/abstract screening. This screening excluded 120 articles that were clearly unrelated to the topic or did not meet the required publication types. Subsequently, 30 articles were selected for full-text review. Detailed full-text assessment excluded 15 articles for the following reasons: two due to insufficient or incomplete diagnostic data (TP, FP, FN, TN); nine because they did not utilize neuroimaging-based ML models; three as they did not specifically address DMG patients with H3K27M mutations; and one non-English article. To ensure comprehensive coverage, we performed supplementary searches by examining the reference lists of two relevant systematic reviews and meta-analyses (22, 23), which finally added one study. After careful checking for and removal of any duplications between database-retrieved and reference list-derived studies, a final set of 16 studies met all inclusion criteria and were included in the meta-analysis (17, 18, 20, 21, 25, 34–44) (Figure 1).

### Study description

A total of 16 studies met the inclusion criteria for this meta-analysis (17, 18, 20, 21, 25, 34–44). These studies, published between 2018 and 2024, collectively enrolled 2,357 patients for internal validation (37 datasets across 16 studies) and 1,792 patients for external validation (12 datasets from 10 studies) (17, 18, 20, 21, 35, 37, 38, 41, 43, 44). All studies employed patient-based analysis using imaging data sourced from local hospital radiology systems. Notably, thirteen of the sixteen studies originated from China (18, 20, 21, 34, 35, 37–44). All included studies were retrospective in design, with two incorporating both retrospective and prospective data collection components (37, 38). The H3K27M mutation status was ultimately determined using immunohistochemistry (IHC) alone in eight studies (17, 20, 21, 25, 36–38, 44), DNA sequencing alone in three studies (35, 40, 41), and a combination of both methods in three studies (18, 42, 43). The remaining two studies did not report the method used to confirm H3K27M mutation status (34, 39).

In terms of methodology, ten studies developed image-only models for predicting H3K27M status (17, 18, 20, 25, 34, 35, 37–39, 44), while six utilized image-clinical integrated models (21, 36, 40–43). Fifteen studies employed MRI as the primary imaging modality; only the study by Yuan et al. utilized PET/CT (20). For model development and validation, data splitting methods included random splitting (8/16 studies) (18, 20, 21, 25, 34, 37, 40, 42), k-fold cross-validation (5/16 studies) (35, 36, 39, 41, 43), and stratified splitting (17, 44) or time series splitting (38) in the remaining studies. Tumor region segmentation was predominantly manual (12/16 studies), performed by experienced radiologists (18, 21, 25, 34–36, 39–44); the remaining studies used semi-automatic methods (17, 37, 38). Notably, Yuan et al. implemented fully automated segmentation without manual input (20). Table 1 and Supplementary Tables 6–9 summarize the study designs, patient cohorts, and methodological features.

**TABLE 1:** Baseline characteristics of patients included in the meta-analysis.

Author	Year	Country	Study design	Location of diffuse midline gliomas	Reference standard	Analysis	Patients per set			No. of H3K27M mutation patients
							Training	Internal validation	External validation	
Guo et al. (34)		China	Retro	NA	NA	PB	72	30	NA	Training:19, Internal validation:8
Huang et al. (35)		China	Retro	NA	DNA sequencing	PB	200	200	35	Training:108, Internal validation:108, External validation:18
Indoria et al. (17)		India	Retro	Thalamus, Brainstem, Cerebellum, Posterior third ventricle, Corpus callosum, Intraventricular, Hypothalamus, Pineal, Septal	IHC	PB	93	31	52	Training:52, Internal validation:17, External validation:33
Jung et al. (36)		South Korea	Retro	Spinal cord	IHC	PB	41	41	NA	Training:24, Internal validation: 24
Kandemirli et al. (25)		USA, Argentina, Turkey	Retro	Thalamus, Pons, Posterior fossa structures	IHC	PB	76	33	NA	Training:32, Internal validation:18
Li et al. (38)		China	Retro, Pro	Spinal cord	IHC	PB	67	30	28	Training:31, Internal validation:14, External validation:16
Li et al. (38)		China	Retro, Pro	Corpus callosum, Thalamus, Brainstem, Spinal cord	IHC	PB	315	130	77	Training:207, Internal validation:72, External validation:36
Pan et al. (40)		China	Retro	Midbrain, Medulla oblongata, Pons	DNA sequencing	PB	106	45	NA	Training:63, Internal validation: 28
Liu et al. (39)		China	Retro	Pons	NA	PB	55	55	NA	Training:35, Internal validation: 35
Su et al. (18)		China	Retro	Thalamus, Brainstem, Spinal cord	IHC, DNA sequencing	PB	75	25	22	Training:30, Internal validation:10, External validation:10
Su et al. (41)		China	Retro	Diencephalon, Brainstem, Cerebellum	DNA sequencing	PB	55	55	13	Training:23, Internal validation:23, External validation:6
Wu et al. (42)		China	Retro	Thalamus, Brainstem, Third ventricle	IHC, DNA sequencing	PB	76	31	NA	Training:56, Internal validation:23
Xiao et al. (43)		China	Retro	Midbrain, Pons, Medulla	IHC, DNA sequencing	PB	117	117	27	Training:80, Internal validation:80, External validation:9
Yang et al. (21)		China	Retro	Midbrain, Pons, Medulla	IHC	PB	93	40	27	Training:56,Internal validation:24, External validation:18
Yuan et al. (20)		China	Retro	Thalamus, Midbrain, Pons, Medulla, Multiple Regions	IHC	PB	90	19	21	Training:45, Internal validation:8, External validation:113
Zhuo et al. (44)		China	Retro	Midbrain, Pons, Medulla	IHC	PB	64	17	29	Training:46, Internal validation:12, External validation:116

Retro, retrospective; Pro, prospective; PB, patient-based; NA, not available; IHC, immunohistochemistry; H3K27M, histone H3 lysine 27 methionine.

### Quality assessment

**FIGURE 2:**
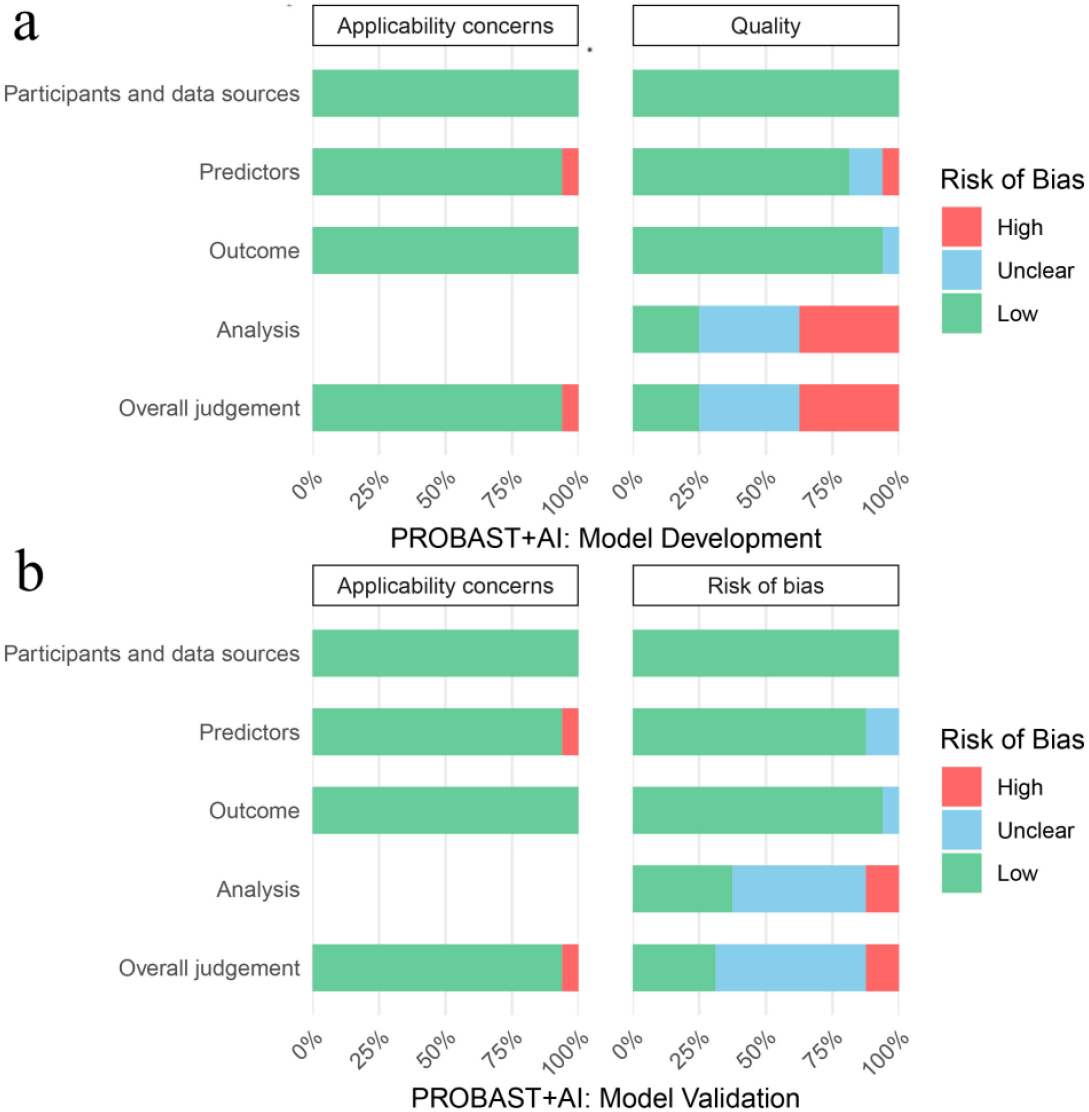
Risk of bias and applicability concerns of the included studies using PROBAST+AI. **(a)** PROBAST+AI model development. **(b)** PROBAST+AI model validation. PROBAST, prediction model risk of bias assessment tool. AI, artificial intelligence.

Risk of bias and applicability concerns were systematically assessed using the PROBAST+AI tool; results are illustrated in Figure 2 and detailed in Supplementary Tables 3, 4. For model development, overall risk of bias was rated as high in 6/16 studies (37.5%), unclear in 6/16 (37.5%), and low in 4/16 (25%). In terms of concerns about applicability, only 1/16 study (6.3%) was rated high risk, while 15/16 (93.8%) were low risk. For model evaluation, overall risk of bias was categorized as high in 2/16 studies (12.5%), unclear in 9/16 (56.3%), and low in 5/16 (31.3%). As for applicability concerns, high risk was observed in only 1/16 study (6.3%), with low risk in the remaining 15/16 (93.8%).

Overall, most studies demonstrated low risk in key domains, with high-risk ratings constituting a minority, indicating acceptable methodological quality for the meta-analysis. The certainty of evidence, assessed using the GRADE framework (Supplementary Table 5), ranged from very low to moderate, reflecting considerable variability and limited strength of evidence.

### Diagnostic performance of different ML algorithms in internal and external validation datasets

**FIGURE 3:**
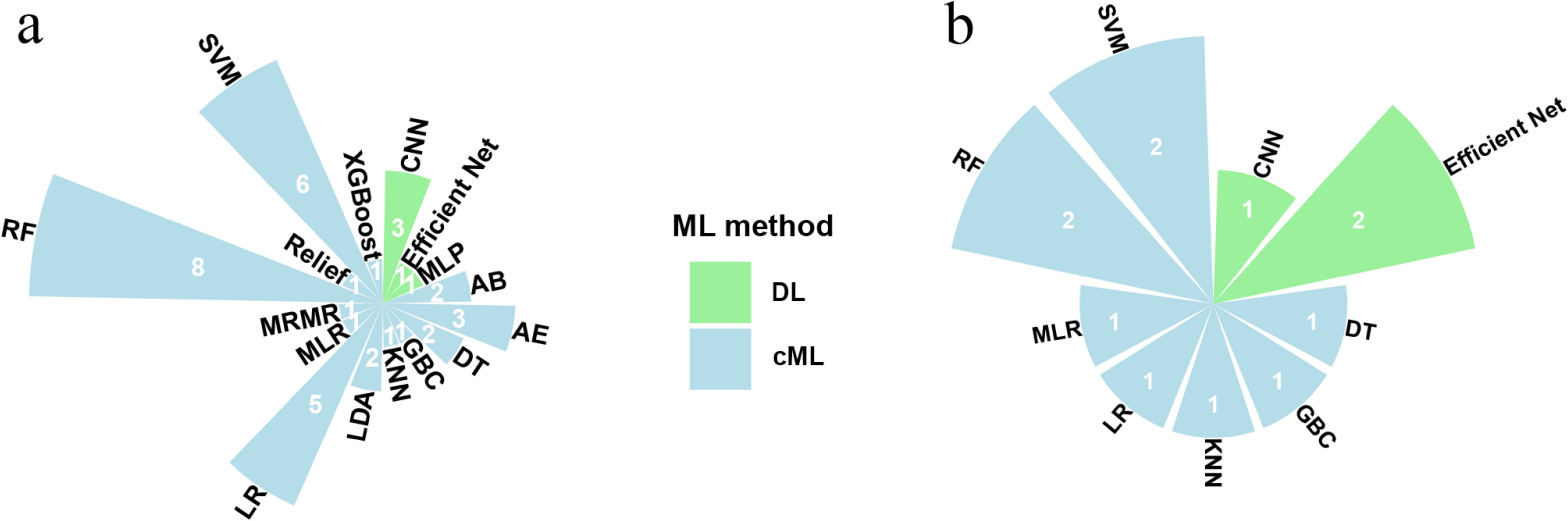
Radar chart of the number of studies using different ML models in neuroimaging for predicting H3K27M mutations in diffuse midline glioma for internal validation set **(a)** and external validation set **(b)**. ML machine learning; cML conventional machine learning; DL deep learning; H3K27M, H3 lysine 27 to methionine.

Analysis of the 37 internal validation datasets revealed that five utilized DL methods and 34 utilized cML methods. Two datasets adopted hybrid approaches combining DL and cML and were therefore counted in both categories for descriptive purposes. Among the DL datasets, CNNs were the most common, utilized in 3/5 (60%) datasets. Within the ML datasets, random forest (RF) and support vector machine (SVM) were the predominant algorithms, comprising 8/34 (23.5%) and 6/34 (17.6%) datasets (Figure 3). Among the algorithms evaluated in the internal validation sets, models based on EfficientNet achieved the highest sensitivity, CNNs-based models achieved the highest specificity, and SVM-based models achieved the highest AUC (Table 2).

**TABLE 2:** Subgroup analysis of different ML algorithms for internal and external validation sets.

ML algorithms	Internal validation datasets				External validation datasets			
	Studies, n	Sensitivity (95%CI)	Specificity (95%CI)	AUC (95%CI)	Studies, n	Sensitivity (95%CI)	Specificity (95%CI)	AUC (95%CI)
AB	2	0.95 (0.79–0.99)	0.79 (0.55–0.92)	NA	NA	NA	NA	NA
AE	3	0.95 (0.84–0.99)	0.48 (0.31–0.66)	NA	NA	NA	NA	NA
Relief	1	0.62 (0.24–0.91)	0.91 (0.71–0.99)	NA	NA	NA	NA	NA
LR	5	0.97 (0.89–0.99)	0.65 (0.47–0.79)	0.92 (0.89–0.94)	1	0.75 (0.48–0.93)	0.83 (0.52–0.98)	NA
RF	7	0.69 (0.50–0.83)	0.86 (0.77–0.92)	0.88 (0.84–0.90)	2	0.82 (0.67–0.91)	0.69 (0.49–0.84)	NA
DT	2	0.89 (0.67–0.97)	0.79 (0.59–0.91)	NA	1	0.82 (0.65–0.93)	0.63 (0.38–0.84)	NA
SVM	5	0.89(0.74–0.96)	0.84(0.72–0.91)	**0.93(0.90–0.95)**	2	0.88(0.73–0.96)	0.82(0.60–0.93)	NA
LDA	2	0.97(0.84–0.99)	0.58(0.37–0.77)	NA	NA	NA	NA	NA
CNN	1	0.87(0.79–0.93)	**0.93(0.86–0.98)**	NA	1	0.78(0.52–0.94)	**0.94(0.71–1.00)**	NA
KNN	1	0.88(0.64–0.99)	0.71(0.42–0.92)	NA	1	0.61(0.42–0.77)	0.63(0.38–0.84)	NA
XGBoost	1	0.72(0.47–0.90)	0.73(0.45–0.92)	NA	NA	NA	NA	NA
EfficientNet	1	**0.98(0.90–1.00)**	0.83(0.66–0.93)	NA	2	**0.94(0.80–0.99)**	0.83(0.68–0.92)	NA
MLP	1	0.89(0.65–0.99)	0.83(0.61–0.95)	NA	NA	NA	NA	NA
CNN, SVM	1	0.97(0.85–1.00)	0.05(0.00–0.25)	NA	NA	NA	NA	NA
CNN, RF	1	0.94(0.81–0.99)	0.20(0.06–0.44)	NA	NA	NA	NA	NA
GBC	1	0.60(0.26–0.88)	0.93(0.68–1.00)	NA	1	0.80(0.44–0.97)	0.92(0.62–1.00)	NA
MRMR	1	0.83(0.61–0.95)	0.88(0.47–1.00)	NA	NA	NA	NA	NA
MLR	1	0.93(0.84–0.97)	0.76(0.59–0.88)	NA	1	0.78(0.40–0.97)	0.89(0.65–0.99)	NA

AUC, area under curve; NA, not available; AB, adaboost; AE, autoencoder; LR, logistic regression; RF, random forest; DT, decision tree; SVM, support vector machine; LDA, linear discriminant analysis; CNN, convolutional neural network; KNN, k-nearest neighbors; XGBoost, extreme gradient boosting; MLP, multilayer perceptron; GBC, gradient boosting classifier; MRMR, minimum redundancy maximum relevance; MLR, multivariable logistic regression. Bold values indicate the highest value among the algorithms.

Evaluation of the 12 external validation datasets showed that three applied DL and nine applied ML. Within the DL datasets utilizing external validation, the EfficientNet architecture was applied in 2/3 (66.7%) datasets. Among the ML datasets, both RF and SVM were each utilized in 2/9 (22.2%) datasets (Figure 3). For the external validation sets, models employing EfficientNet demonstrated the highest sensitivity, while models employing CNN demonstrated the highest specificity among the evaluated algorithms (Table 2).

### Internal and external validation sets for MRI-based ML models

**FIGURE 4:**
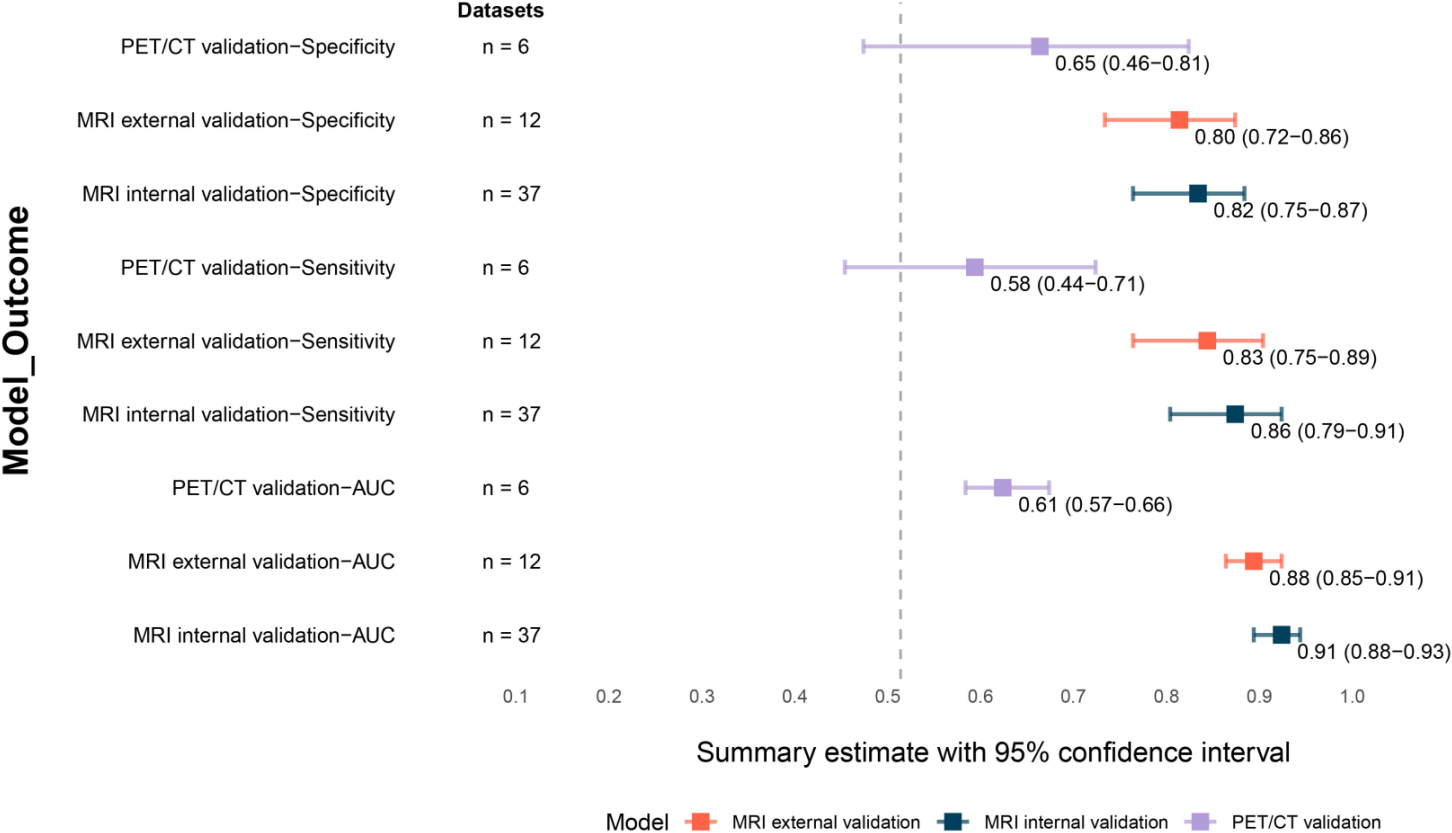
Forest plots of diagnostic performance across MRI-based ML internal validation set, MRI-based ML external validation set, and PET/CT overall validation set. MRI, magnetic resonance imaging. PET/CT, positron emission tomography/computed tomography.

For the internal validation set, pooled estimates demonstrated that MRI-based ML models achieved a sensitivity of 0.86 (95% confidence interval [CI]: 0.79–0.91) and a specificity of 0.82 (95% CI: 0.75–0.87), both with low certainty. The pooled AUC was 0.91 (95% CI: 0.88–0.93) (Figure 4). Correspondingly, for the external validation set, the sensitivity was 0.83 (95% CI: 0.75–0.89) and the specificity was 0.80 (95% CI: 0.72–0.86), also with low certainty; the pooled AUC was 0.88 (95% CI: 0.85–0.91) (Figure 4). No statistically significant differences between internal and external validation sets in terms of sensitivity (*Z* = 0.63, *P* = 0.52), specificity (*Z* = 0.42, *P* = 0.67), or AUC values (*Z* = 1.50, *P* = 0.13).

### Heterogeneity

**FIGURE 5:**
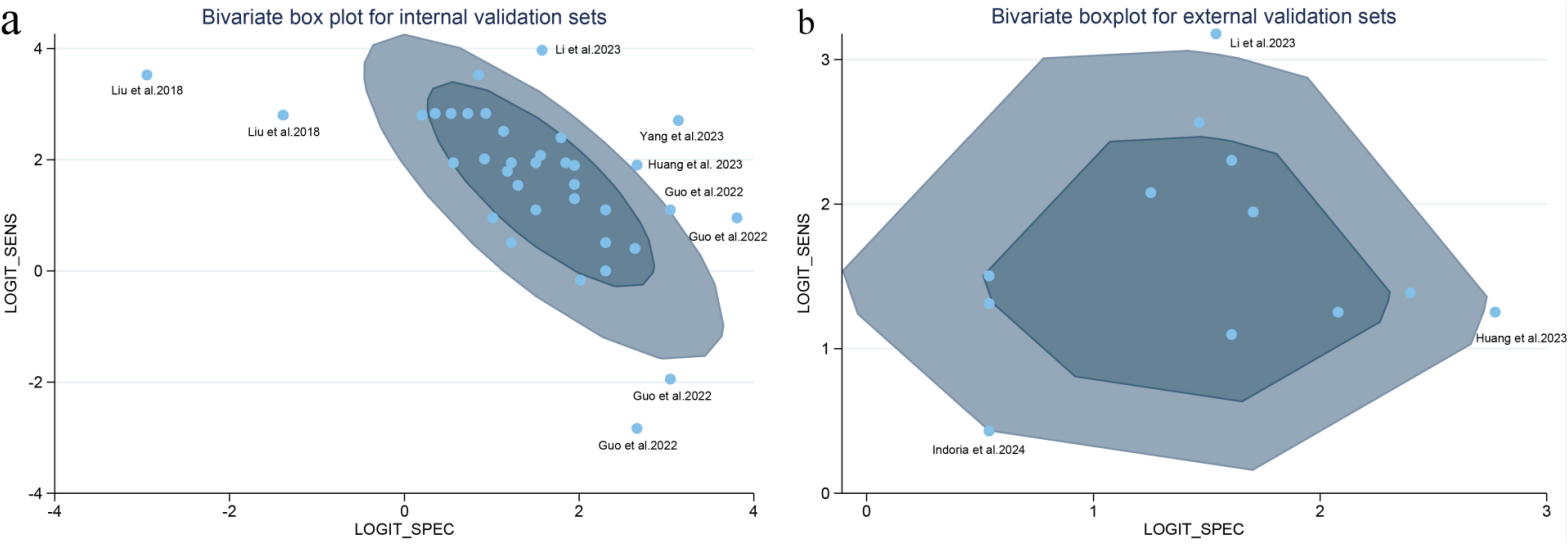
Bivariate box plots of diagnostic performance for MRI-based ML internal validation set **(a)** and MRI external validation set **(b)**. MRI, magnetic resonance imaging.

Substantial heterogeneity was observed for both sensitivity (*I*^2^ = 78.17%) and specificity (*I*^2^ = 79.44%; *I*^2^ > 50% indicates substantial heterogeneity) in internal validation set. To explore the potential sources of this heterogeneity, meta-regression analyses were performed. The results indicated that differences in the reference standard (*P* < 0.001), ML method (*P* = 0.01), and MRI strength (*P* < 0.001) were significant sources of heterogeneity (Table 3). Outlier analysis using bivariate boxplots identified studies by Liu et al., Li et al., Yang et al., Huang et al., and Guo et al. as discrete values, identifying them as potential sources of heterogeneity within this cohort (21, 34, 35, 38, 39) (Figure 5a).

**TABLE 3:** Subgroup analysis and meta-regression based on MRI internal validation set.

Subgroup	Studies, n	Sensitivity (95%CI)	Meta–regression *P*-value	Specificity (95%CI)	Meta-regression *P*-value
Location of DMG			0.51		0.17
Brain	16	0.91 (0.87–0.95)		0.74 (0.62–0.86)	
Spinal cord	3	0.71 (0.51–0.92)		0.88 (0.72–1.00)	
Reference standard			0.17		< 0.001
IHC	10	0.86 (0.78–0.94)		0.83 (0.77–0.88)	
DNA sequencing	3	0.87 (0.76–0.98)		0.90 (0.85–0.95)	
ML model			0.58		0.16
Image-only	32	0.86 (0.80–0.92)		0.82 (0.75–0.88)	
Image and Clinical	5	0.82 (0.66–0.99)		0.85 (0.71–0.99)	
ML method			0.06		0.01
Conventional machine learning	32	0.83 (0.76–0.90)		0.83 (0.78– 0.87)	
Deep learning	3	0.94 (0.85–1.00)		0.88 (0.79–0.98)	
Regions of interest			0.06		0.25
Manually	31	0.84 (0.78–0.91)		0.82 (0.76–0.89)	
Semi-automatic or automatic	6	0.90 (0.81–1.00)		0.82 (0.67–0.96)	
MRI strength			0.29		< 0.001
1.5 T and 3.0 T	9	0.87 (0.78–0.97)		0.86 (0.79–0.93)	
3.0 T	23	0.83 (0.75–0.91)		0.84 (0.79–0.89)	

DMG, diffuse midline glioma; IHC, immunohistochemistry; ML, machine learning; MRI, magnetic resonance imaging; T, tesla.

In contrast, for the external validation set, heterogeneity was moderate for sensitivity (*I*^2^ = 41.26%) and low for specificity (*I*^2^ = 26.46%; *I*^2^ < 50% generally indicates low to moderate heterogeneity). Nevertheless, meta-regression suggested that MRI strength (*P* = 0.02), ML method (*P* = 0.01), and the reference standard (*P* = 0.05) remained significant contribute to the heterogeneity in the external validation studies (Supplementary Table 10). Bivariate boxplot analysis identified studies by Li et al., Indoria et al., and Huang et al. as outliers (17, 35, 38) for external validation sets (Figure 5b).

### Clinical application and publication bias

**FIGURE 6:**
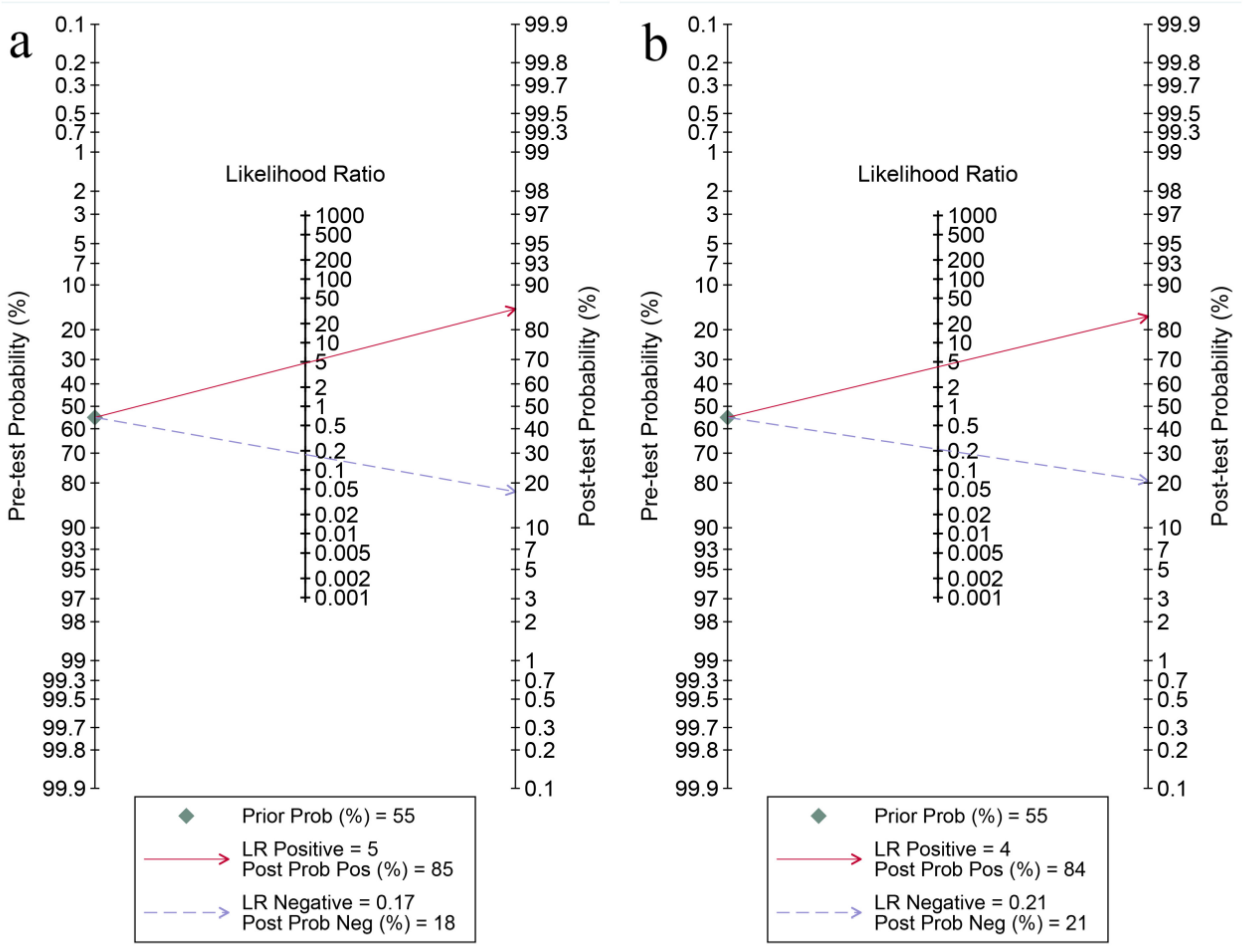
Fagan nomograms of diagnostic probability for MRI-based ML internal validation set **(a)** and MRI external validation set **(b)**. MRI, magnetic resonance imaging.

**FIGURE 7:**
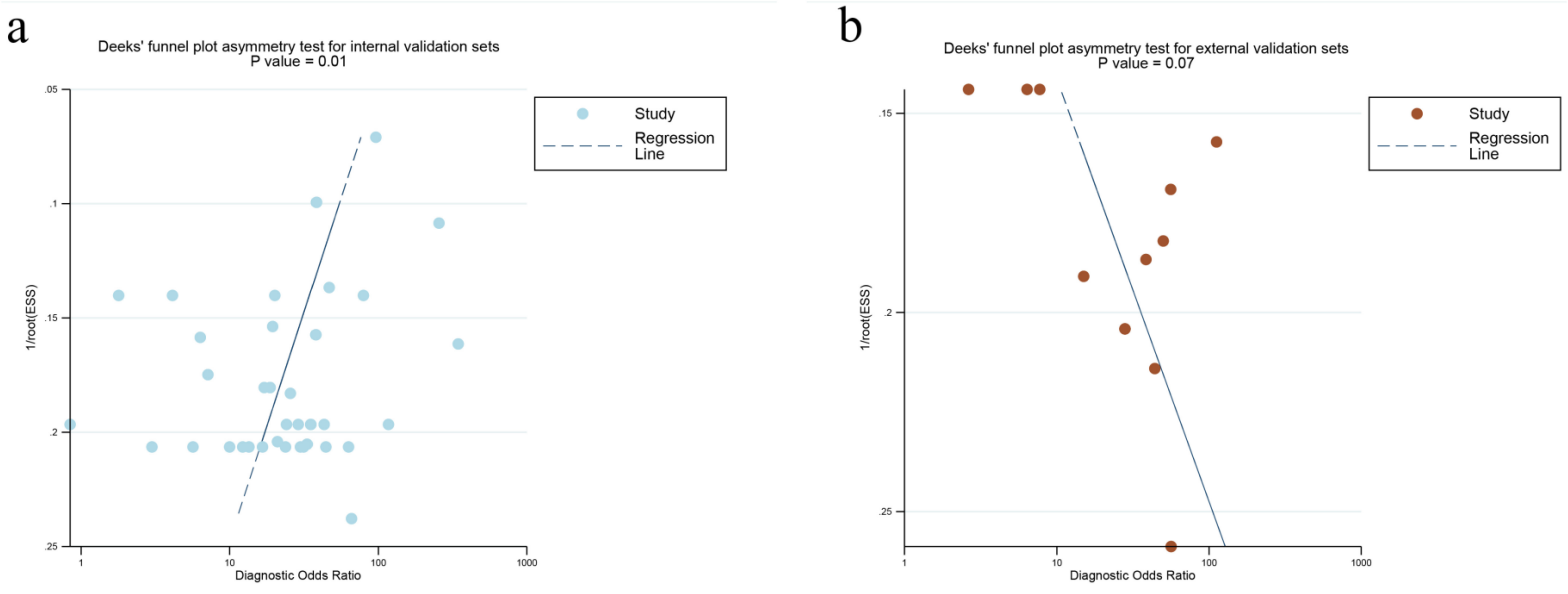
Deek’s funnel plots for publication bias in MRI-based DL internal validation set **(a)** and MRI external validation set **(b)**. MRI, magnetic resonance imaging.

Fagan nomograms were constructed using a pre-test probability of 55%, based on the median prevalence across included studies. For internal validation, the positive and negative likelihood ratios were 85 and 18%, respectively (Figure 6a); for external validation, they were 84% and 21% (Figure 6b). These results support the diagnostic utility of the models under varying prevalence conditions. Publication bias was assessed using Deeks’ funnel plot. Significant bias was observed in internal validation (*P* = 0.01), but not in external validation (*P* = 0.07) (Figures 7a,b).

### ML model based on PET/CT

Pooled sensitivity for PET/CT-based ML models was 0.58 (95% CI: 0.44–0.71; moderate certainty), while specificity reached 0.65 (95% CI: 0.46–0.81; low certainty). The summary AUC was 0.61 (95% CI: 0.57–0.66) (Figure 4). Comparative analysis demonstrated statistically significant differences between MRI-based and PET/CT-based models for sensitivity (*Z* = 3.71; *P* < 0.01) and AUC (*Z* = 11.42; *P* < 0.01), but not for specificity (*Z* = 1.80; *P* = 0.07).

## Discussion

Our meta-analysis indicates that MRI-based ML models achieve consistently strong diagnostic discrimination for predicting H3K27M mutation status in DMG, with performance remaining broadly consistent in external validation cohorts (37, 38). This suggests that ML can leverage imaging phenotypes that are difficult to quantify visually—such as radiomic, morphologic, and microstructural patterns embedded in routine and advanced MRI sequences—which may reflect the underlying molecular alteration (35, 41). Nevertheless, between-study heterogeneity and the overall low-to-moderate certainty of evidence indicate that reproducibility is not guaranteed and should be verified in well-designed prospective multicenter settings.

When comparing imaging modalities, the available evidence suggests MRI-based ML may outperform PET/CT-based ML for H3K27M prediction, plausibly because MRI provides richer anatomic and tissue-contrast information (e.g., diffusion, FLAIR, perfusion) that better captures tumor phenotype (17, 45, 46). However, it is crucial to note that this comparative analysis was primarily informed by a single included PET/CT study, substantially limiting the robustness of any modality-level conclusions. Future work should evaluate PET/CT-based ML in larger, protocol-standardized cohorts (including tracer harmonization) and ideally in head-to-head designs to establish whether PET adds complementary value beyond MRI (20).

With respect to modeling strategy, DL appeared to show more favorable generalization than cML in the available externally validated datasets, which aligns with DL’s ability to learn hierarchical, spatially preserved representations directly from images without handcrafted feature engineering (35, 39). At the same time, this comparison is vulnerable to imbalance and pipeline confounding: the DL subgroup is small, while the cML subgroup is dominated by radiomics-based workflows that may not represent the best-performing non-DL methods. Accordingly, the DL-cML contrast should be interpreted as hypothesis-generating, and future studies should compare optimized DL and cML pipelines under the same data, preprocessing, and validation framework (20, 38, 41).

Reference standard selection has direct implications for both model training and evaluation. The higher specificity observed in sequencing-referenced studies may reflect lower label noise than IHC-referenced labels. While effective for detecting H3K27M protein expression, IHC may misclassify non-mutant tumors as positive when aberrant histone modifications mimic the target epitope (47). When ML models are trained using IHC as the reference standard, they may inadvertently “learn” from mislabeled data, which can reduce accuracy in identifying true negative cases and thereby lower specificity. In contrast, DNA sequencing precisely identifies H3K27M mutations at the genetic level and may provide a more reliable reference for model development and evaluation, supporting improved specificity and generalizability (47). These findings support considering DNA sequencing as the preferred reference standard for future ML model development and benchmarking (18, 47, 48).

In contrast to the meta-analysis by Habibi et al. (22), which synthesized 13 studies (*n* = 1,510) evaluating MRI radiomics-based ML models and meta-analyzed 6 studies, our meta-analysis expands the evidence base by including 16 studies with a larger total sample and, importantly, by synthesizing internal and external validation results separately to better characterize performance under independent evaluation. Methodologically, we further introduced the PROBAST+AI tool (28), enabling comprehensive risk-of-bias assessment at both the model development and validation stages—addressing a key standardization gap. In contrast, Habibi et al.’s assessment was limited to the QUADAS-2 tool, applied only to original diagnostic studies (22). Notably, we provide quantitative evidence supporting the potential superiority of MRI-based ML over PET/CT for H3K27M prediction, although this conclusion is constrained by sparse PET/CT data. This multimodal comparison was not included in the prior review by Habibi et al. (22).

While the narrative synthesis by Haddadi Avval et al. (23) systematically outlined AI’s expanding role in DMG, the cited high-performance studies (e.g., Guo et al., AUC 0.97) were predominantly single-center with small samples (*n* ≤ 102) and lacked independent external validation (34). In our external validation cohorts (*n* = 1,792), MRI-based ML models demonstrated consistently robust performance (AUC 0.88), effectively mitigating the potential overestimation bias often observed in smaller, single-center studies. Collectively, our work enhances the evidence base for clinical translation by providing extensive validation and multimodal comparison. These efforts complement and extend prior literature, elevating both the level of evidence and practical utility for ML-driven molecular diagnostics in DMG.

The substantial heterogeneity observed across the included studies may compromise the precision and generalizability of pooled sensitivity and specificity estimates for MRI-based ML models predicting H3K27M mutation status in DMG. To investigate potential sources of heterogeneity in internal and external validation cohorts, we conducted meta-regression and bivariate boxplot analyses. Meta-regression suggested that variability may be partially attributable to the choice of reference standard, the type of ML method (DL vs. cML), and MRI field strength. Bivariate boxplots analysis further identified specific studies as potential outliers influencing the internal validation results, including Liu et al. (39), Li et al. (38), Guo et al. (34), Yang et al. (21), and Huang et al. (35). For external validation cohort, meta-regression indicated similar sources of heterogeneity, with Li et al. (38), Indoria et al. (17), and Huang et al. (35) identified as potential outliers via boxplot analysis. It is important to note that this pronounced heterogeneity likely stems from additional factors beyond those formally tested. Additional sources may include variations in patient demographics (e.g., age distribution), tumor characteristics (e.g., WHO grade composition), geographic and institutional settings, data preprocessing pipelines, validation strategies, sample sizes, and key model development specifics—such as feature selection, hyperparameter tuning, and algorithm implementation (22). The cumulative effect of these methodological and clinical differences likely accounts for a substantial portion of the inter-study performance variation. Despite this heterogeneity, the aggregated evidence consistently indicates the promising diagnostic potential of ML-assisted neuroimaging for the non-invasive prediction of H3K27M status in DMG (22, 23).

Our meta-analysis suggests that MRI-based ML models demonstrate promisingly predictive performance for H3K27M mutations status in DMG across validation datasets. However, the acknowledged heterogeneity and very low-to-moderate certainty of evidence necessitate a cautious interpretation of these pooled estimates. While these findings indicate the potential for such tools to eventually aid noninvasive molecular stratification and clinical decision support within specialized care pathways, substantial work is needed before real-world deployment. Key translational gaps include reproducibility across institutions and scanner platforms, standardization of image acquisition and preprocessing, and robust external validation and regulatory-grade evaluation frameworks. Any clinical integration should initially position ML as a decision-support adjunct rather than a replacement for standard diagnostic procedures, which are informed by a holistic assessment of clinical, histopathological, and molecular data (23). Future development should prioritize multimodal integration and interpretability, yet these efforts must concurrently address the substantial challenges of data harmonization, privacy-preserving analytics, and interoperability with existing hospital systems (49). Ultimately, bridging these gaps in reproducibility, standardization, and regulatory science will be paramount for the safe, effective, and equitable translation of promising AI tools into accountable neuro-oncological practice (49).

Several limitations of this meta-analysis warrant careful consideration when interpreting the results. First, not all included studies utilized DNA sequencing as the gold standard for detecting H3K27M mutations. This variability in reference standards may have introduced heterogeneity and potentially biased diagnostic accuracy estimates. Although subgroup analyses and meta-regressions by reference standard were performed, these findings suggest that this factor may particularly affect specificity. Consequently, future studies are encouraged to adopt DNA sequencing as the preferred reference standard to enhance comparability and accuracy (21, 22). Second, to enable a comprehensive comparison of diagnostic performance across diverse algorithms and model types, we extracted all eligible models reported in each primary study. Although methodologically necessary, this approach introduced the risk of patient-level data overlap or duplicate model inclusion from the same cohort, thereby increasing the potential for selection bias. Third, the geographical imbalance of included studies, with a predominance of Chinese cohorts (13/16), limits the generalizability of our findings. Differences in tumor biology, imaging protocols, and healthcare systems across regions may affect ML model performance and applicability. Therefore, large-scale, multinational validation studies are needed to confirm the robustness and transportability of these results (20, 22). Fourth, significant publication bias was detected in the internal validation meta-analysis (*P* = 0.01), suggesting potential overestimation of pooled diagnostic accuracy due to the underrepresentation of smaller studies with negative results. Although external validation showed no significant bias, the overall evidence strength should be interpreted with consideration for potential small-study effects. Fifth, for some studies, diagnostic contingency table data were indirectly estimated from reported ROC curves using the Youden index. While this is a common practice when raw data are unavailable, it may introduce measurement inaccuracies, particularly in studies with small sample sizes or class imbalance, potentially affecting the precision of the pooled diagnostic accuracy estimates.

## Conclusion

In conclusion, MRI-based ML demonstrates high accuracy and generalizability for non-invasive H3K27M prediction in DMG, seemingly outperforming current PET/CT-based ML, although modality-level conclusions are constrained by sparse PET/CT data. DL-based approaches and sequencing-based reference standards appear to contribute to improved performance, supporting the potential clinical utility of MRI-based ML for molecular stratification.

## Data Availability

The original contributions presented in the study are included in the article/Supplementary material, further inquiries can be directed to the corresponding author.
