# Supplementary material for: Machine learning in neuroimaging for predicting H3K27M mutations in diffuse midline gliomas: a systematic review and meta-analysis
Source: Front Med (Lausanne). 2026 Mar 25;13:1746840. doi: 10.3389/fmed.2026.1746840 (PMC13056679; doi:10.3389/fmed.2026.1746840)
Supplement: Supplementary file 1 [file Data_Sheet_1.pdf]

Table of Contents

Supplementary Table 1 Preferred Reporting Items for Systematic Reviews and Meta-Analyses of Diagnostic Test Accuracy (PRISMA-DTA) Checklist. 2

Supplementary Table 2 Search strategy in PubMed, Embase, Cochrane and Web of Science. 4

Supplementary Table 3 Summary of inclusion criteria using the PITROS framework. 6

Supplementary Table 4 Risk of bias assessment (PROBAST+AI) model development based on seven domains. 7

Supplementary Table 5 Risk of bias assessment (PROBAST+AI) model evaluation based on seven domains. 9

Supplementary Table 6 GRADE scoring assessments in all of the pooled outcomes. 11

Supplementary Table 7 Technical aspects of included studies. 13

Supplementary Table 8 MRI imaging data summary of included studies 14

Supplementary Table 10 Diagnostic performance data of the internal validation set and external validation sets for PET/CT. 18

Supplementary Table 11 Subgroup analysis and meta-regression based on MRI external validation set 19

Supplementary Table 1 Preferred Reporting Items for Systematic Reviews and Meta-Analyses of Diagnostic Test Accuracy (PRISMA-DTA) Checklist.

TITLE/ABSTRACT			Reported on page #
Title	1	Identify the report as a systematic review (+/−meta-analysis) of diagnostic test accuracy (DTA) studies	1
Abstract	2	See the PRISMA for Abstracts checklist.	2
INTRODUCTION			
Rationale	3	Describe the rationale for the review in the context of what is already known	2
Clinical role of index test	D1	State the scientific and clinical background, including the intended use and clinical role of the index test, and if applicable, the rationale for minimally acceptable test accuracy (or minimum difference in accuracy for comparative design)	3
Objectives	4	Provide an explicit statement of question(s) being addressed in terms of participants, index test(s), and target condition(s)	4
METHODS			
Protocol and registration	5	Indicate if a review protocol exists, if and where it can be accessed (e.g., Web address), and, if available, provide registration information including registration number.	4
Eligibility criteria	6	Specify study characteristics (participants, setting, index test(s), reference standard(s), target condition(s), and study design) and report characteristics (e.g., years considered, language, publication status) used as criteria for eligibility, giving rationale.	5
Information sources	7	Describe all information sources (e.g., databases with dates of coverage, contact with study authors to identify additional studies) in the search and date last searched.	6
Search	8	Present full search strategies for all electronic databases and other sources searched, including any limits used, such that they could be repeated.	7
Selection process	9	State the process for selecting studies (i.e., screening, eligibility, included in systematic review, and, if applicable, included in the meta-analysis).	7-8
Data collection process	10	Describe method of data extraction from reports (e.g., piloted forms, independently, in duplicate) and any processes for obtaining and confirming data from investigators.	7-8
Definitions for data extraction	11	Provide definitions used in data extraction and classifications of target condition(s), index test(s), reference standard(s) and other characteristics (e.g., study design, clinical setting).	8
Risk of bias and applicability	12	Describe methods used for assessing risk of bias in individual studies and concerns regarding the applicability to the review question.	9
Diagnostic accuracy measures	13	State the principal diagnostic accuracy measure(s) reported (e.g., sensitivity, specificity) and state the unit of assessment (e.g., per-patient, per-lesion).	
Synthesis of results	12	Describe methods of handling data, combining results of studies and describing variability between studies. This could include, but is not limited to: a) handling of multiple definitions of target condition. b) handling of multiple thresholds of test positivity, c) handling multiple index test readers, d) handling of indeterminate test results, e) grouping and comparing tests, f) handling of different reference	9

		standards	
Meta-analysis	D2	Report the statistical methods used for meta-analyses, if performed.	9
Additional analyses	16	Describe methods of additional analyses (e.g., sensitivity or subgroup analyses, meta-regression), if done, indicating which were pre-specified.	10
RESULTS			
Study selection	17	Provide numbers of studies screened, assessed for eligibility, included in the review (and included in meta-analysis, if applicable) with reasons for exclusions at each stage, ideally with a flow diagram.	10
Study characteristics	18	For each included study provide citations and present key characteristics including: a) participant characteristics (presentation, prior testing), b) clinical setting, c) study design, d) target condition definition, e) index test, f) reference standard, g) sample size, h) funding sources	11
Risk of bias in studies	19	Present evaluation of risk of bias and concerns regarding applicability for each study.	12
Results of individual studies	20	For each analysis in each study (e.g., unique combination of index test, reference standard, and positivity threshold) report 2x2 data (TP, FP, FN, TN) with estimates of diagnostic accuracy and confidence intervals, ideally with a forest or receiver operator characteristic (ROC) plot.	13
Synthesis of results	22	Describe test accuracy, including variability; if meta-analysis was done, include results and confidence intervals.	14
Additional analysis	23	Give results of additional analyses, if done (e.g., sensitivity or subgroup analyses, meta-regression; analysis of index test: failure rates, proportion of inconclusive results, adverse events).	15-16
DISCUSSION			
Summary of evidence	24	Summarize the main findings including the strength of evidence.	16-20
Limitations	25	Discuss limitations from included studies (e.g., risk of bias and concerns regarding applicability) and from the review process (e.g., incomplete retrieval of identified research).	21-23
Conclusions	26	Provide a general interpretation of the results in the context of other evidence. Discuss implications for future research and clinical practice (e.g., the intended use and clinical role of the index test).	24
FUNDING			
Funding	26	For the systematic review, describe the sources of funding and other support and the role of the funders.	25

Salameh, Jean-Paul et al. “Preferred reporting items for systematic review and meta-analysis of diagnostic test accuracy studies (PRISMA-DTA): explanation, elaboration, and checklist.” *BMJ (Clinical research ed.)* vol. 370 m2632. 14 Aug. 2020, doi:10.1136/bmj.m2632

Supplementary Table 2 Search strategy in PubMed, Embase, Cochrane and Web of Science.

Database	Search strategy	Filters and Limits	Number of Studies
PubMed	((("Artificial Intelligence"[Mesh] OR "Machine Learning"[Mesh] OR "Deep Learning"[Mesh] OR "Neural Networks, Computer"[Mesh] OR (AI[Title/Abstract] OR "machine learning"[Title/Abstract] OR "deep learning"[Title/Abstract] OR "neural network*"[Title/Abstract] OR "computer vision"[Title/Abstract] OR "natural language processing"[Title/Abstract] OR "predictive model*"[Title/Abstract] OR "algorithm*"[Title/Abstract])) OR "Radiographic Image Interpretation, Computer-Assisted"[Mesh] OR "Image Processing, Computer-Assisted"[Mesh] OR (radiomic*[Title/Abstract] OR "radiomics"[Title/Abstract] OR "texture analysis"[Title/Abstract] OR "imaging biomarker*"[Title/Abstract] OR "quantitative imaging"[Title/Abstract] OR "radiomic feature*"[Title/Abstract] OR "CT radiomic*"[Title/Abstract] OR "MRI radiomic*"[Title/Abstract] OR "PET radiomic*"[Title/Abstract]))<br><br>AND (("Diffuse Intrinsic Pontine Glioma"[Mesh] OR "Brain Stem Neoplasms"[Mesh] OR "Glioma"[Mesh] OR "diffuse midline glioma*"[Title/Abstract] OR "DMG"[Title/Abstract] OR "diffuse intrinsic pontine glioma*"[Title/Abstract] OR "DIPG"[Title/Abstract] OR "brainstem glioma*"[Title/Abstract] OR "pontine glioma*"[Title/Abstract] OR "thalamic glioma*"[Title/Abstract] OR "spinal cord glioma*"[Title/Abstract] OR "midline glioma*"[Title/Abstract] OR "pediatric high-grade glioma*"[Title/Abstract])))<br><br>AND (("H3K27M"[Title/Abstract] OR "H3 K27M"[Title/Abstract] OR "H3.3K27M"[Title/Abstract] OR "H3F3A K27M"[Title/Abstract] OR "K27M mutation"[Title/Abstract] OR "Histone H3 K27M"[Title/Abstract] OR "H3K27M mutant"[Title/Abstract] OR "H3K27M alteration"[Title/Abstract] OR "H3K27M-positive"[Title/Abstract]))	No restrictions applied regarding date range, language, or publication type.	39
Embase	('artificial intelligence'/exp OR 'machine learning'/exp OR 'deep learning'/exp OR ai:ti,ab OR 'machine learning':ti,ab OR 'deep learning':ti,ab OR 'neural network*':ti,ab OR 'computer vision':ti,ab OR 'natural language processing':ti,ab OR 'predictive model*':ti,ab OR algorithm*:ti,ab OR 'radiomics'/exp OR 'texture analysis'/exp OR 'computer assisted diagnosis'/exp OR radiomic*:ti,ab OR 'radiomics':ti,ab OR 'texture analysis':ti,ab OR 'imaging biomarker*':ti,ab OR 'quantitative imaging':ti,ab OR 'radiomic feature*':ti,ab OR 'ct radiomic*':ti,ab OR 'mri radiomic*':ti,ab OR 'pet radiomic*':ti,ab)<br><br>AND ('brain stem tumor'/exp OR 'glioma'/exp OR 'pontine glioma'/exp OR 'diffuse midline glioma*':ti,ab OR dmg:ti,ab OR 'diffuse intrinsic pontine glioma*':ti,ab OR dipg:ti,ab OR	No restrictions applied regarding date range, language, or publication type.	136

	'brainstem glioma*':ti,ab OR 'pontine glioma*':ti,ab OR 'thalamic glioma*':ti,ab OR 'spinal cord glioma*':ti,ab OR 'midline glioma*':ti,ab OR 'pediatric high-grade glioma*':ti,ab)<br><br>AND (h3k27m AND gene:ti,ab OR h3k27m:ti,ab OR 'h3 k27m':ti,ab OR 'h3.3k27m':ti,ab OR 'h3f3a k27m':ti,ab OR 'k27m mutation':ti,ab OR 'histone h3 k27m':ti,ab OR 'h3k27m mutant':ti,ab OR 'h3k27m alteration':ti,ab OR 'h3k27m-positive':ti,ab)		
Web of Science	TS=("Artificial Intelligence" OR "Machine Learning" OR "Deep Learning" OR "Neural Networks, Computer" OR AI OR "machine learning" OR "deep learning" OR "neural network*" OR "computer vision" OR "natural language processing" OR "predictive model*" OR "algorithm*" OR "Radiographic Image Interpretation, Computer-Assisted" OR "Image Processing, Computer-Assisted" OR radiomic* OR "radiomics" OR "texture analysis" OR "imaging biomarker*" OR "quantitative imaging" OR "radiomic feature*" OR "CT radiomic*" OR "MRI radiomic*" OR "PET radiomic*") AND TS=(("Diffuse Intrinsic Pontine Glioma" OR "Brain Stem Neoplasms" OR "Glioma") OR ("diffuse midline glioma*" OR "DMG" OR "diffuse intrinsic pontine glioma*" OR "DIPG" OR "brainstem glioma*" OR "pontine glioma*" OR "thalamic glioma*" OR "spinal cord glioma*" OR "midline glioma*" OR "pediatric high-grade glioma*")) AND TS=("H3K27M" OR "H3 K27M" OR "H3.3K27M" OR "H3F3A K27M" OR "K27M mutation" OR "Histone H3 K27M" OR "H3K27M mutant" OR "H3K27M alteration" OR "H3K27M-positive")	No restrictions applied regarding date range, language, or publication type.	59
Cochrane Library	((Artificial Intelligence):ti,ab,kw OR (Machine Learning):ti,ab,kw OR (Deep Learning):ti,ab,kw OR (Neural Networks, Computer):ti,ab,kw OR AI OR (machine learning):ti,ab,kw OR (deep learning):ti,ab,kw OR (neural network*):ti,ab,kw OR (computer vision):ti,ab,kw OR (natural language processing):ti,ab,kw OR (predictive model*):ti,ab,kw OR (algorithm*):ti,ab,kw OR (Radiographic Image Interpretation, Computer-Assisted):ti,ab,kw OR (Image Processing, Computer-Assisted):ti,ab,kw OR radiomic* OR (radiomics):ti,ab,kw OR (texture analysis):ti,ab,kw OR (imaging biomarker*):ti,ab,kw OR (quantitative imaging):ti,ab,kw OR (radiomic feature*):ti,ab,kw OR (CT radiomic*):ti,ab,kw OR (MRI radiomic*):ti,ab,kw OR (PET radiomic*):ti,ab,kw) AND ((Diffuse Intrinsic Pontine Glioma):ti,ab,kw OR (Brain Stem Neoplasms):ti,ab,kw OR (Glioma):ti,ab,kw OR (diffuse midline glioma*):ti,ab,kw OR (DMG):ti,ab,kw OR (diffuse intrinsic pontine glioma*):ti,ab,kw OR (DIPG):ti,ab,kw OR (brainstem glioma*):ti,ab,kw OR (pontine glioma*):ti,ab,kw OR (thalamic glioma*):ti,ab,kw OR (spinal cord glioma*):ti,ab,kw OR (midline glioma*):ti,ab,kw OR (pediatric high-grade glioma*):ti,ab,kw) AND ((H3K27M):ti,ab,kw OR (H3 K27M):ti,ab,kw OR (H3.3K27M):ti,ab,kw OR (H3F3A K27M):ti,ab,kw OR (K27M mutation):ti,ab,kw OR (Histone H3 K27M):ti,ab,kw OR (H3K27M mutant):ti,ab,kw OR (H3K27M alteration):ti,ab,kw OR (H3K27M-positive):ti,ab,kw)	No restrictions applied regarding date range, language, or publication type.	2

Supplementary Table 3 Summary of inclusion criteria using the PITROS framework.

Inclusion Criteria	Details
Participants (P)	Studies including patients with pathologically confirmed diffuse midline glioma (DMG) with explicit reporting of H3K27M mutation status (mutant versus wild-type) were eligible.
Index test (I)	Eligible studies utilized artificial intelligence techniques (machine learning, deep learning, or radiomics) for neuroimaging analysis (MRI, PET/CT) to predict H3K27M mutation.
Target conditions (T)	H3K27M mutation-positive status comprised the positive group, and H3K27M wild-type comprised the negative group.
Reference standard (R)	Pathological confirmation, typically via biopsy, served as the reference standard for diagnosis.
Outcomes (O)	Primary outcomes were sensitivity, specificity, and area under the receiver operating characteristic curve (AUC) in internal and/or external validation cohorts.
Settings (S)	Both retrospective and prospective studies were considered, including those based on local hospitals.

Supplementary Table 4 Risk of bias assessment (PROBAST+AI) model development based on seven domains.

Author, year		Quality				Applicability concerns			Overall judgement	
		Participants and data sources [a]	Predictors [b]	Outcome [c]	Analysis [d]	Participants and data sources [e]	Predictors [f]	Outcome [g]	Quality [h]	Applicability concerns [i]
Guo et al.	2022	L	L	L	L	L	L	L	L	L
Huang et al.	2023	L	U	L	U	L	L	L	U	L
Indoria et al.	2024	L	L	L	U	L	L	L	U	L
Jung et al.	2019	L	H	L	H	L	L	L	H	L
Kandemirli et al.	2021	L	L	L	L	L	L	L	L	L
Li et al.	2023	L	L	L	H	L	L	L	H	L
Li et al.	2023	L	L	L	U	L	L	L	U	L
Pan et al.	2019	L	L	L	U	L	L	L	U	L
Liu et al.	2018	L	L	L	H	L	L	L	H	L
Su et al.	2020	L	L	U	H	L	L	L	H	L
Su et al.	2022	L	L	L	H	L	H	L	H	H
Wu et al.	2022	L	L	L	U	L	L	L	U	L
Xiao et al.	2024	L	U	L	U	L	L	L	U	L
Yang et al.	2023	L	L	L	L	L	L	L	L	L
Zhuo et al.	2021	L	L	L	H	L	L	L	H	L
Yuan et al.	2024	L	L	L	L	L	L	L	L	L

Abbreviation: PROBAST+AI, Prediction model Risk of Bias Assessment Tool + AI, L low; H high; U unclear.

Footnote: Signaling questions are rated as "yes" (Y), "probably yes" (PY), "probably no" (PN), "no" (N), "no information" (NI), and in some cases "not applicable" (NA). All signaling questions are phrased in such a way that "yes" or "probably yes" indicates a low risk of bias. Any signaling questions rated as "no" or "probably no" indicate a potential high risk of bias in that domain. If there are no "no" or "probably no" ratings, but "no information" (NI) is present, the risk of bias in that domain is classified as unclear.

a. Participants and data sources

1.1 Were appropriate data sources used?

1.2 Was an appropriate study design used?

1.3 Did the in- and exclusions of study participants result in a representative dataset?

b. Predictors

2.1 Were predictors defined and assessed in a similar way for all participants?

2.2 Was any pre-processing of predictors similar for all participants?

2.3 Were predictor assessments made without knowledge of outcome data?

2.4 Were the predictors included in the model available at the time the model was intended to be used?

c. Outcome

3.1 Were outcomes defined and assessed appropriately?

3.2 Were outcomes defined and assessed in a similar way for all participants?

3.3 Were outcome assessments made without use or knowledge of predictor data?

3.4 Was the time interval between predictor assessment and outcome assessment appropriate?

d. Analysis

4.1 Was there evidence that the sample size was reasonable?

4.2 Were continuous and categorical predictors handled appropriately?

4.3 Were participants with missing or censored data handled appropriately in the analysis?

4.4 If methods to address class imbalance were used, was the model or the model predictions recalibrated?

4.5 Were methods used to address potential model overfitting?

e. Participants and data sources

Concern that the (data of the) included participants do not match the review question or the assessor's intended use of the prediction model.

f. Predictors

Concern that the definition, pre-processing, assessment, or timing of assessment of the predictors in the model do not match the review question or the assessor's intended use.

g. Outcome

Concern that the outcome, its definition, assessment, or timing of assessment do not match the review question or the assessor's intended use.

h. Quality

Low risk: If all four domains were rated low concern regarding quality.

High risk: If at least one domain was rated high concern regarding quality .

Unclear: If at least one domain was rated unclear concern regarding quality and no domains were rated high concern.

i. Applicability concerns

Low risk: If all three domains were rated low concern for applicability.

High risk: If at least one domain was rated high concern for applicability.

Unclear: If at least one domain was rated unclear concern for applicability and no domains were rated high concern.

Supplementary Table 5 Risk of bias assessment (PROBAST+AI) model evaluation based on seven domains.

Author, year		Risk of bias				Applicability concerns			Overall judgement	
		Participants and data sources [a]	Predictors [b]	Outcome [c]	Analysis [d]	Participants and data sources [e]	Predictors [f]	Outcome [g]	Risk of bias [h]	Applicability concerns
Guo et al.	2022	L	L	L	L	L	L	L	L	L
Huang et al.	2023	L	U	L	U	L	L	L	U	L
Indoria et al.	2024	L	L	L	U	L	L	L	U	L
Jung et al.	2019	L	L	L	H	L	L	L	H	L
Kandemirli et al.	2021	L	L	L	L	L	L	L	L	L
Li et al.	2023	L	L	L	U	L	L	L	U	L
Li et al.	2023	L	L	L	U	L	L	L	U	L
Pan et al.	2019	L	L	L	U	L	L	L	U	L
Liu et al.	2018	L	L	L	H	L	L	L	H	L
Su et al.	2020	L	L	U	L	L	L	L	U	L
Su et al.	2022	L	L	L	U	L	H	L	U	H
Wu et al.	2022	L	L	L	U	L	L	L	U	L
Xiao et al.	2024	L	U	L	U	L	L	L	U	L
Yang et al.	2023	L	L	L	L	L	L	L	L	L
Zhuo et al.	2021	L	L	L	L	L	L	L	L	L
Yuan et al.	2024	L	L	L	L	L	L	L	L	L

Abbreviation: PROBAST+AI, Prediction model Risk of Bias Assessment Tool + AI, L low; H high; U unclear.

Footnote: Signaling questions are rated as "yes" (Y), "probably yes" (PY), "probably no" (PN), "no" (N), "no information" (NI), and in some cases "not applicable" (NA). All signaling questions are phrased in such a way that "yes" or "probably yes" indicates a low risk of bias. Any signaling questions rated as "no" or "probably no" indicate a potential high risk of bias in that domain. If there are no "no" or "probably no" ratings, but "no information" (NI) is present, the risk of bias in that domain is classified as unclear.

a. Participants and data sources

1.1 Were appropriate data sources used?

1.2 Was an appropriate study design used?

1.3 Did the in- and exclusions of study participants result in a representative dataset?

b. Predictors

2.1 Were predictors defined and assessed in a similar way for all participants?

2.2 Was any pre-processing of predictors similar for all participants?

2.3 Were predictor assessments made without knowledge of outcome data?
2.4 Were the predictors included in the model available at the time the model was intended to be used?
c. Outcome
3.1 Were outcomes defined and assessed appropriately?
3.2 Were outcomes defined and assessed in a similar way for all participants?
3.3 Were outcome assessments made without use or knowledge of predictor data?
3.4 Was the time interval between predictor assessment and outcome assessment appropriate?
d. Analysis
4.1 Was model evaluation based on only apparent performance avoided?
4.2 Was there evidence that the sample size was reasonable?
4.3 Were participants with missing or censored data handled appropriately in the analysis?
4.4 If methods to address class imbalance were used, was the evaluation done in a dataset without imbalance correction?
4.5 If data splitting was done to create training and test datasets, was there evidence that data leakage was avoided?
4.6 If resampling methods were used to evaluate model performance, were all model development steps replicated in the resampling process?
4.7 Was the predictive performance of the model evaluated appropriately, e.g., calibration, discrimination, and net benefit?
e. Participants and data sources
Concern that the (data of the) included participants do not match the review question or the assessor's intended use of the prediction model.
f. Predictors
Concern that the definition, pre-processing, assessment, or timing of assessment of the predictors in the model do not match the review question or the assessor's intended use.
g. Outcome
Concern that the outcome, its definition, assessment, or timing of assessment do not match the review question or the assessor's intended use.
h. Risk of bias
Low risk: If all four domains were rated low risk of bias.
High risk: If at least one domain was rated high risk of bias.
Unclear: If at least one domain was rated unclear risk of bias and no domains were rated high risk of bias.
i. Applicability concerns
Low risk: If all three domains were rated low concern for applicability.
High risk: If at least one domain was rated high concern for applicability.
Unclear: If at least one domain was rated unclear concern for applicability and no domains were rated high concern

Supplementary Table 6 GRADE scoring assessments in all of the pooled outcomes.

Dataset	Outcome	Risk of Bias[a]	Inconsistency[b]	Indirectness[c]	Imprecision[d]	Publication Bias[e]	Total Downgrade	Final Rating
Internal validation dataset for MRI	Sensitivity	1	0	1	1	1	4	Very low
	Specificity	1	0	1	1	1	4	Very low
External validation dataset for MRI	Sensitivity	1	0	1	1	0	3	Very low
	Specificity	1	0	1	1	0	3	Very low
Validation datasets for PET/CT	Sensitivity	0	0	0	1	0	1	Moderate
	Specificity	0	1	0	1	0	2	Low

a. Risk of Bias

Assessed using tools like PROSBAST+AI to evaluate study design and methodology.

Downgrade by 1 level if at least one study has a high risk of bias.

b. Inconsistency

Measured using I^2 statistics to assess heterogeneity across studies.

Downgrade by 1 level if $I^2 > 50\%$ and the source of heterogeneity cannot be explained.

If the heterogeneity is identified (e.g., through meta-regression), no downgrade is applied.

c. Indirectness

Evaluates whether study populations, interventions, or outcomes differ from the target research question.

Patient Indirectness: Downgrade if the included population significantly deviates from the target population (e.g., specific subgroups).

Outcome Indirectness: Downgrade if inconsistent outcome measures are used, such as estimating sensitivity/specificity indirectly from ROC-based Youden Index.

d. Imprecision

Assessed based on confidence intervals (CIs) and sample size sufficiency.

For sensitivity/specificity: Downgrade if 95% CI width > 0.20 or if CIs cross clinical thresholds (e.g., 0.80).

Sample size threshold: Total sample < 500 or positive/negative cases < 100.

e. Publication Bias

Evaluated using Deek’s Funnel Plot or similar tools.
Downgrade by 1 level if the funnel plot shows significant asymmetry or if P-value < 0.05 in Deek’s tests.

Supplementary Table 7 Technical aspects of included studies.

Author	Year	Data source	ML method	ML model	Data splitting method	Type of neuroimaging
Guo et al.	2022	Local hospital	Machine learning	Image-only model	Random split	MRI
Huang et al.	2023	Local hospital	Deep learning	Image-only model	5-fold cross validation	MRI
Indoria et al.	2024	Local hospital	Machine learning	Image-only model	Stratified data splitting	MRI
Jung et al.	2019	Local hospital	Machine learning	Image-clinical model	10-fold cross validation	MRI
Kandemirli et al.	2021	Local hospital	Machine learning	Image-only model	Random split	MRI
Li et al.	2023	Local hospital	Machine learning	Image-only model	Random split	MRI
Li et al.	2023	Local hospital	Deep learning	Image-only model	Time Series Splitting	MRI
Pan et al.	2019	Local hospital	Machine learning	Image-clinical model	Random split	MRI
Liu et al.	2018	Local hospital	Deep learning, Machine learning	Image-only model	5-fold cross validation	MRI
Su et al.	2020	Local hospital	Machine learning	Image-only model	Random split	MRI
Su et al.	2022	Local hospital	Machine learning	Image-clinical model	5-fold cross validation	MRI
Wu et al.	2022	Local hospital	Machine learning	Image-clinical model	Random split	MRI
Xiao et al.	2024	Local hospital	Machine learning	Image-clinical model	5-fold cross validation	MRI
Yang et al.	2023	Local hospital	Machine learning	Image-clinical model	Random split	MRI
Yuan et al.	2024	Local hospital	Deep learning	Image-only model	Random split	^{11}C-Methionine PET/CT, ^{18}F-Fluoroethyltyrosine PET/CT
Zhuo et al.	2021	Local hospital	Machine learning	Image-only model	Stratified data splitting	MRI

MRI magnetic resonance imaging; PET/CT positron emission tomography/computed tomography.

Supplementary Table 8 MRI imaging data summary of included studies

Author	Year	Scanner Modality (System)	Feature extraction(software)	Evaluation Time	Regions of interest (ROI)	Magnetic field strength
Guo et al.	2022	MAGNETOM Verio, Skyra, Prisma	FeAture Explore(V 0.4.2)	Pre-operative	Manually	3.0 T
Huang et al.	2023	Philips Achieva, GE MR 750 W, Siemens Healthcare, uMR780, Achieva	LabelMe(V 3.16.2)	Pre-operative	Manually	3.0 T, 1.5 T
Indoria et al.	2024	Aera (Siemens Medical Systems, Erlangen, Germany), Ingenia (Philips Medical Systems, Best, Netherlands)	PyRadiomics	Pre-operative	Semi-automatic	3.0 T, 1.5 T
Jung et al.	2019	Magnetom Expert, Magnetom Avanto, Magnetom Trio A Tim, Genesis Signa, Excite, Intera, Achieva	NA	Pre-operative	Manually	3.0 T, 1.5 T, 1.0 T
Kandemirli et al.	2021	NA	PyRadiomics(V3.0)	Pre-operative	Manually	3.0 T, 1.5 T
Li et al.	2023	SIGNA HDe, Discovery 750, Ingenia CX, Verio, Magnetom Trio, Magnetom Prisma	FAE(V0.5.2)	Pre-operative	Semi-automatic	3.0 T, 1.5 T
Li et al.	2023	GE Signa HDxt, GE Discovery MR750, Siemens MAGNETOM Verio, Siemens MAGNETOM Prisma, Philips Ingenia CX, GE Signa Excite, Siemens MAGNETOM Avanto, Siemens TrioTim, Siemens Skyra, Philips Achieva, GE Signa Discovery MR750, Siemens MAGNETOM TrioTim, Siemens MAGNETOM Essenza	Deep Learning Network	Pre-operative	Semi-automatic	3.0 T, 1.5 T
Pan et al.	2019	GE Discovery MR750	3D Slicer(V4.1)	Pre-operative	Manually	3.0 T
Liu et al.	2018	NA	CNN automatic, 3D Slicer	Pre-operative	Manually	NA
Su et al.	2020	Siemens, Philips Achieva, GE MR 750W, Toshiba Medical Systems, Alltech Medical Systems	PyRadiomics	Pre-operative	Manually	3.0 T, 1.5 T
Su et al.	2022	Skyra	ITK-SNAP(V3.6.0), FSL, LCModel	Pre-operative	Manually	3.0 T
Wu et al.	2022	Ingenia, GE Signa HD	PyRadiomics	Pre-operative	Manually	3.0 T
Xiao et al.	2024	Ingenia CX	PyRadiomics (V2.2.0)	Pre-operative	Manually	3.0 T
Yang et al.	2023	Ingenia CX	PyRadiomics (V3.0.1)	Pre-operative	Manually	3.0 T
Yuan et al.	2024	Ingenia, Verio, Signa HDxt	Pyradiomics	Pre-operative	Fully automatic	NA
Zhuo et al.	2021	Ingenia CX	FAE(V0.3.6)	Pre-	Manually	3.0 T

operative

FAE Feature Analysis Explorer; ITK-SNAP Insight Segmentation and Registration Toolkit - Segmentation and Navigation Assistant Program; FSL Functional Magnetic Resonance Imaging of the Brain Software Library; LCModel Linear Combination Model.

Supplementary Table 9 Diagnostic performance data of the internal validation set and external validation sets for MRI.

Author	Year	MRI sequence	ML method	ML algorithms	Internal validation				External validation			
					TP	FP	FN	TN	TP	FP	FN	TN
		T2WI	ML	AB	6	0	2	22	NA	NA	NA	NA
		T1WI	ML	AE	8	9	0	13	NA	NA	NA	NA
		FLAIR	ML	Relief	5	2	3	20	NA	NA	NA	NA
		CE-T1WI	ML	LR	8	6	0	16	NA	NA	NA	NA
		ADC	ML	RF	0	1	8	21	NA	NA	NA	NA
		SWI	ML	DT	7	3	1	19	NA	NA	NA	NA
		CBV	ML	AE	7	8	1	14	NA	NA	NA	NA
		CBF	ML	LR	7	4	1	18	NA	NA	NA	NA
		T2WI, CE-T1WI	ML	SVM	4	2	4	20	NA	NA	NA	NA
Guo et al.	2022	T2WI, CE-T1WI, ADC	ML	AE	5	5	3	17	NA	NA	NA	NA
		T2WI, CE-T1WI, SWI	ML	LDA	6	4	2	18	NA	NA	NA	NA
		T2WI, CE-T1WI, CBF	ML	RF	1	1	7	21	NA	NA	NA	NA
		T2WI, CE-T1WI, ADC, SWI	ML	SVM	6	2	2	20	NA	NA	NA	NA
		T2WI, CE-T1WI, ADC, CBF	ML	LR	6	1	2	21	NA	NA	NA	NA
		T2WI, CE-T1WI, SWI, CBF	ML	LDA	8	7	0	15	NA	NA	NA	NA
		cMRI	ML	LR	8	8	0	14	NA	NA	NA	NA
		aMRI	ML	AB	7	5	1	17	NA	NA	NA	NA
Huang et al.	2023	CE-T1WI, T2WI	DL	CNN	94	6	14	86	14	1	4	16
		T2WI	ML	DT	14	3	3	11	27	7	6	12
Indoria et al.	2024	T2WI	ML	RF	15	4	2	10	26	7	7	12
		T2WI	ML	KNN	15	4	2	10	20	7	13	12
Jung et al.	2019	T1WI, T2WI, CE-T1WI	ML	RF	11	2	13	15	NA	NA	NA	NA
Kandemirli et al.	2021	T1WI, T2WI, CE-T1WI, ADC, FLAIR	ML	XGBoost	13	4	5	11	NA	NA	NA	NA
Li et al.	2023	T2WI	ML	LR	11	2	3	14	12	2	4	10
		T2WI	DL	EfficientNet-B0	53	6	1	29	24	3	1	14
Li et al.	2023								10	4	1	20
		T2WI	DL	MLP	16	4	2	19	NA	NA	NA	NA
Pan et al.	2019	T1WI, T2WI, CE-T1WI	ML	RF	24	4	4	13	NA	NA	NA	NA

Liu et al.	2018	T1w-MPRAGE	DL+ML	CNN, SVM	34	19	1	1	NA	NA	NA	NA
		T1w-MPRAGE	DL+ML	CNN, RF	33	16	2	4	NA	NA	NA	NA
		T1w-MPRAGE	ML	SVM	33	9	2	11	NA	NA	NA	NA
		T1w-MPRAGE	ML	RF	34	6	1	14	NA	NA	NA	NA
Su et al.	2020	FLAIR	ML	GBC	6	1	4	14	8	1	2	11
Su et al.	2022	T2W_FS, FLAIR, T1w-MPRAGE, DWI, DTI, PWI, MRS	ML	RF	20	4	3	28	6	1	0	6
Wu et al.	2022	FLAIR, DWI, ADC, CE-T1WI	ML	MRMR	19	1	4	7	NA	NA	NA	NA
Xiao et al.	2024	T1WI, DWI, DTI, DKI	ML	MLR	74	9	6	28	7	2	2	16
Yang et al.	2023	T1WI, T2WI, CE-T1WI, DWI	ML	SVM	15	1	1	23	16	2	2	7
Zhuo et al.	2021	APTw	ML	SVM	11	1	1	6	14	2	2	11

SFR Stone Free Rate; ML Machine learning; DL Deep learning; TP true positive; TN true negative; FP false positive; FN false positive; NA not available; AB AdaBoost; AE Autoencoder; LR Logistic Regression; RF Random Forest; DT Decision Tree; SVM Support Vector Machine; LDA Linear Discriminant Analysis; CNN Convolutional Neural Network; KNN K-Nearest Neighbors; XGBoost eXtreme Gradient Boosting; MLP Multilayer Perceptron; GBC Gradient Boosting Classifier; MRMR Minimum Redundancy Maximum Relevance; MLR Multivariable Logistic Regression; T1WI T1-Weighted Imaging; T2WI T2-Weighted Imaging; FLAIR Fluid-Attenuated Inversion Recovery; CE-T1WI Contrast-Enhanced T1-weighted Imaging; ADC Apparent Diffusion Coefficient; SWI Susceptibility Weighted Imaging; CBV Cerebral Blood Volume; CBF Cerebral Blood Flow; cMRI Conventional MRI(T2WI, T1WI, FLAIR and CE-T1WI); aMRI Advanced MRI(ADC, SWI, CBV and CBF); T1w-MPRAGE T1-Weighted Magnetization Prepared Rapid Gradient Echo; T2W_FS T2-Weighted Fast Spin Echo with Fat Suppression, DTI Diffusion Tensor Imaging; PWI Perfusion Weighted Imaging; MRS Magnetic Resonance Spectroscopy; DKI Diffusion Kurtosis Imaging; APTw Amide Proton Transfer-weighted.

Supplementary Table 10 Diagnostic performance data of the internal validation set and external validation sets for PET/CT.

Author	Year	ML method	ML algorithms	Internal validation				External validation			
				TP	FP	FN	TN	TP	FP	FN	TN
Yuan et al.	2024	DL	AT-CNN	7	3	1	8	9	2	4	6
		ML	SVM	5	7	3	4	NA	NA	NA	NA
		ML	RF	3	7	5	4	NA	NA	NA	NA
		ML	LR	3	1	5	10	NA	NA	NA	NA
		DL	CNN	4	3	4	8	NA	NA	NA	NA

SFR Stone Free Rate; ML Machine learning; DL Deep learning; TP true positive; TN true negative; FP false positive; FN false positive; NA not available. PET/CT Positron Emission Tomography/Computed Tomography, AT-CNN Assistance Training Convolutional Neural Network; SVM Support Vector Machine; RF Random Forest; LR Logistic Regression; CNN Convolutional Neural Network.

Supplementary Table 11 Subgroup analysis and meta-regression based on MRI external validation set

Subgroup	Studies, n	Sensitivity(95%CI)	Meta-regression *P*-value	Specificity(95%CI)	Meta-regression *P*-value
Location of DMG			0.54		0.10
Brain	9	0.84 (0.76-0.92)		0.77 (0.69-0.86)	
Spinal cord	2	0.83 (0.65-1.00)		0.83 (0.69-0.97)	
Reference standard			0.48		0.05
IHC	8	0.83 (0.75-0.91)		0.75 (0.66-0.83)	
DNA sequencing	2	0.85 (0.68-1.00)		0.92 (0.81-1.00)	
ML model			0.48		0.10
Image-only	10	0.82 (0.76-0.89)		0.78 (0.71-0.86)	
Image & Clinical	2	0.87 (0.70-1.00)		0.89 (0.75-1.00)	
ML method			0.01		0.01
Conventional machine learning	9	0.80 (0.72-0.88)		0.76 (0.68-0.85)	
Deep learning	3	0.89 (0.80-0.98)		0.86 (0.77-0.96)	
Regions of interest			0.18		0.80
Manually	6	0.85 (0.76-0.94)		0.88 (0.81-0.96)	
Semi-automatic or automatic	6	0.81 (0.72-0.89)		0.73 (0.64-0.82)	
MRI strength			0.02		0.02
1.5 T and 3.0 T	8	0.81 (0.73-0.88)		0.78 (0.69-0.86)	
3.0 T	4	0.88 (0.79-0.98)		0.86 (0.75-0.96)	

DMG diffuse midline glioma; IHC immunohistochemistry; AI artificial intelligence; MRI magnetic resonance imaging; T tesla.
